# [Retracted] LTBP2 promotes the migration and invasion of gastric cancer cells and predicts poor outcome of patients with gastric cancer

**DOI:** 10.3892/ijo.2026.5847

**Published:** 2026-01-13

**Authors:** Jun Wang, Wen-Jia Liang, Guang-Tao Min, Hong- Peng Wang, Wei Chen, Nan Yao

Int J Oncol 52: 1886-1898, 2018; DOI: 10.3892/ijo.2018.4356

Following the publication of this paper, it was drawn to the Editor's attention by a concerned reader that certain of the Transwell cell migration and invasion assay data shown in Fig. 5A on p. 1895 were strikingly similar to data in an article written by different authors at different research institutes that had already been accepted for publication in the journal *OncoTargets and Therapy*. Owing to the fact that the contentious data in the above article had already been published prior to its submission to *International Journal of Oncology*, the Editor has decided that this paper should be retracted from the Journal. The authors were asked for an explanation to account for these concerns, but the Editorial Office did not receive a reply. The Editor apologizes to the readership for any inconvenience caused.

